# Effects of crystalloids and colloids on liver and intestine microcirculation and function in cecal ligation and puncture induced septic rodents

**DOI:** 10.1186/1471-230X-12-179

**Published:** 2012-12-17

**Authors:** Martin Alexander Schick, Jobst Tobias Isbary, Tanja Stueber, Juergen Brugger, Jan Stumpner, Nicolas Schlegel, Norbert Roewer, Otto Eichelbroenner, Christian Wunder

**Affiliations:** 1 Department of Anaesthesia and Critical Care, University of Würzburg, Oberdürrbacherstraße 6, Würzburg, 97080, Germany; 2 Department of Anaesthesiology and Critical Care, Clinic of Chemnitz gGmbH, Flemmingstraße 2, Chemnitz, 09116, Germany; 3 Department of General, Visceral, Vascular and Paediatric Surgery (Department of Surgery II), University of Würzburg, Oberdürrbacherstraße 6, Würzburg, 97080, Germany

**Keywords:** Sepsis, Microcirculation, Colloids, HES, Crystalloids

## Abstract

**Background:**

Septic acute liver and intestinal failure is associated with a high mortality. We therefore investigated the influence of volume resuscitation with different crystalloid or colloid solutions on liver and intestine injury and microcirculation in septic rodents.

**Methods:**

Sepsis was induced by cecal ligation and puncture (CLP) in 77 male rats. Animals were treated with different crystalloids (NaCl 0.9% (NaCl), Ringer’s acetate (RA)) or colloids (Gelafundin 4% (Gel), 6% HES 130/0.4 (HES)). After 24 h animals were re-anesthetized and intestinal (n = 6/group) and liver microcirculation (n = 6/group) were obtained using intravital microscopy, as well as macrohemodynamic parameters were measured. Blood assays and organs were harvested to determine organ function and injury.

**Results:**

HES improved liver microcirculation, cardiac index and DO_2_-I, but significantly increased IL-1β, IL-6 and TNF-α levels and resulted in a mortality rate of 33%. Gel infused animals revealed significant reduction of liver and intestine microcirculation with severe side effects on coagulation (significantly increased PTT and INR, decreased haemoglobin and platelet count). Furthermore Gel showed severe hypoglycemia, acidosis and significantly increased ALT and IL-6 with a lethality of 29%. RA exhibited no derangements in liver microcirculation when compared to sham and HES. RA showed no intestinal microcirculation disturbance compared to sham, but significantly improved the number of intestinal capillaries with flow compared to HES. All RA treated animals survided and showed no severe side effects on coagulation, liver, macrohemodynamic or metabolic state.

**Conclusions:**

Gelatine 4% revealed devastated hepatic and intestinal microcirculation and severe side effects in CLP induced septic rats, whereas the balanced crystalloid solution showed stabilization of macro- and microhemodynamics with improved survival. HES improved liver microcirculation, but exhibited significantly increased pro-inflammatory cytokine levels. Crystalloid infusion revealed best results in mortality and microcirculation, when compared with colloid infusion.

## Background

Acute liver failure (ALF) and sepsis share some conformity, e.g. coagulopathy, shock and a high mortality. As a combination of both, the sepsis induced ALF is the lethal duo. Despite of intensive research and improvement of treatment, patients in septic shock as well as patients with ALF show a survival rate of less than 50% [1,2].

The liver and the intestine play a pivotal role in sepsis by modulating immune response due to release and filtering cytokines, bacterial fragments and vasomodulating substances. Hepatic hypoperfusion and microcirculation disturbances lead to contracted sinusoids, swollen sinusoidal endothelial cells and activated Kupffer cells. The hepatic response to this stimulus is a release of toxic, pro-inflammatory and vasoactive substances, which trigger the vicious circle of multi organ failure (MOF) and death [3,4]. The hepatic histopathological changes prominent after septic shock may be due to impaired microcirculation and therefore cellular hypoxia [5]. Hepatic perfusion is clinically difficult to determine and can’t be predicted by systemic hemodynamic parameters. However cardiac output (CO) showed a direct correlation to total hepatic flow [6] but hepatic microcirculation remained heterogeneous with regional flow deficits.

During septic shock in pigs superior mesenteric artery and hepatic microcirculation flow decreased about 50% and hydroxyethylstarch administration improved splanchnic blood flow but not microvascular liver perfusion [4]. In addition repeated administration of hydroxyethylstarch can aggravate portal hypertension, liver failure and sepsis in patients with chronic liver disease [7]. On the other side, Catre and colleagues have demonstrated protective effects of HES 130/0.4 on hepatic reperfusion damage [8]. However volume resuscitation is a cornerstone in the treatment of sepsis and shock. The volume replacement strategies are different between countries and are even different between ICUs in the same hospital [9]. Crystalloids (CL) and natural or artificial colloids (COL) are commonly used to restore fluid balance.

In the last decades controversy results were published and now the German sepsis guidelines 2010 recommended the use of CL in sepsis. Despite of numerous clinical studies there is no evidence for the safety use of third generation HES 130/0.4 in critical ill patients [10]. In contrast there are increasing results of severe side effects of synthetic colloids to kidney function and mortality [11-14]. However little is known about the impact of different CL and COL solutions on the hepatic and intestinal microcirculation in sepsis. Therefore we designed this study to investigate the direct influence of different solutions on liver and intestine microcirculation and function in a rodent sepsis model.

## Methods

### Animals

After animal care committee approval (Laboratory Animal Care and Use Committee of the District of Unterfranken, Germany), which meets applicable European legislation and the standards of the National Institutes of Health as set forth in the Guide for the Care and Use of Laboratory Animals (National Research Council, Washington: National Academy Press, 1996), experiments were performed on 80 male Sprague–Dawley rats (345 ± 30 g bodyweight (BW), Harlan Winkelmann, Germany). All animals were maintained on a standard diet, water ad libitum and 12 h day and night cycles. Animals were not fasted prior and after the CLP procedure.

### Experimental protocol

**Figure 1:**
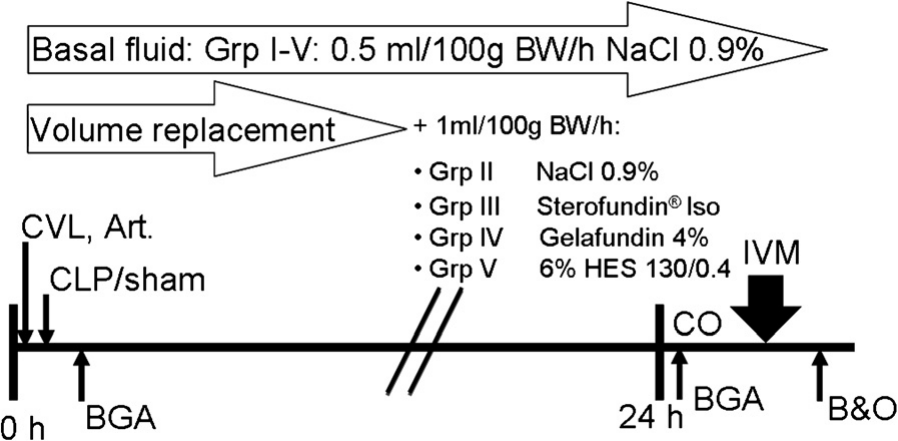
**Experimental setup.** All animals were randomized and analysed in a blinded fashion. The aim was to analyse 6 animals per group for intestinal and liver microcirculation. Since animals died due to the initiated sepsis, additional animals were randomized and analysed.

**Figure 2:**
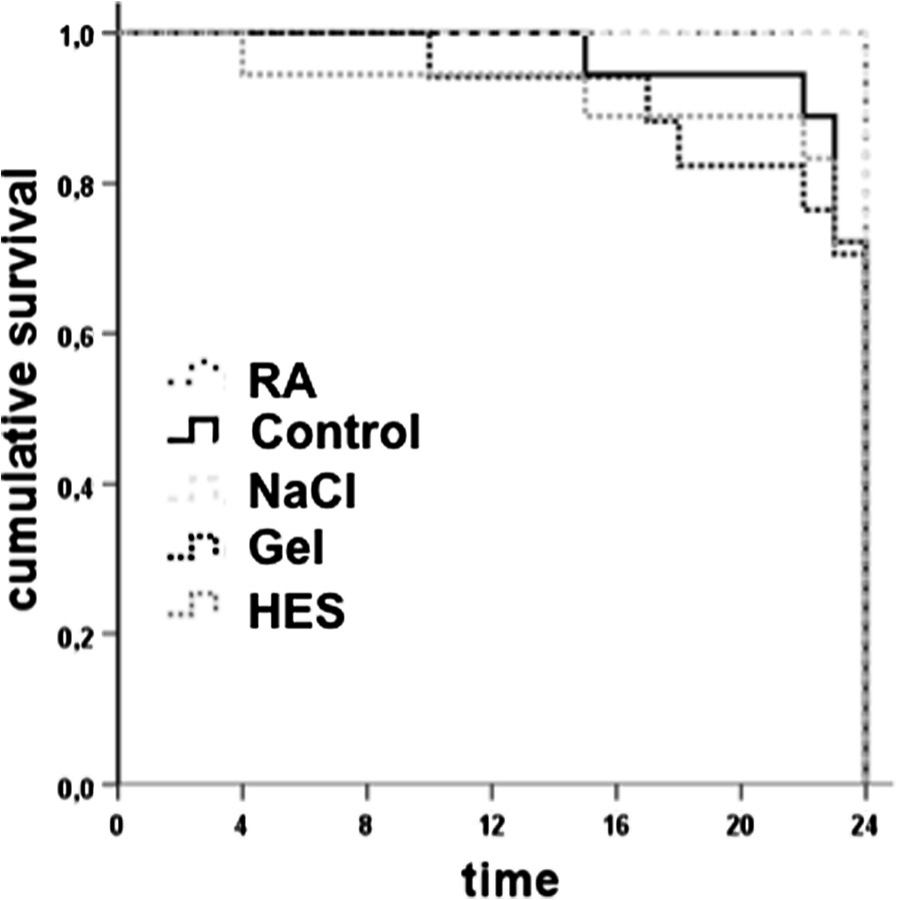
**Survival curves.** Schematic presentation of the experimental trial. Sepsis was performed by coecum ligation and puncture (CLP). Central venous line (CVL) and continuous invasive arterial pressure line (Art.) were implanted and fluid administration was performed (group (Grp), body weight (BW)). Blood gas analyses (BGA) were measured 30 minutes after surgical procedures and after 24 h with cardiac output (CO) calculations. Afterwards data of the liver sinusoidal and intestinal blood flow were collected using intravital microscopy (IVM) and at the end of the experiment blood samples and organs (B&O) were harvested.

Animals were randomized (Figure 1) to receive liver or intestine examination and afterwards to group (grp) I-V (n = 6/ grp), grp I: Sham, grp II: NaCl (NaCl 0,9% Fresenius Kabi, Germany), grpIII: RA (Ringer’s acetate, Sterofundin®ISO B. Braun Melsungen AG, Germany), grp IV: Gel (Gelafundin 4%, B. Braun Melsungen AG, Germany), grp V: HES (6% HES 130/0,4, Voluven®, Fresenius Kabi, Germany), and anesthetized using isoflurane(Forene®, Abbott, Germany)/ nitrous oxide inhalation. The carrier solution of Gel and HES consisted of aqua ad injectabilis (Gel: sodium 154 mval, chloride 120 mval; HES: sodium 154 mval, chloride 154 mval). Body temperature was monitored via a rectal probe during surgery and intravital microscopy. Body temperature was maintained at 37.0 ± 0.5°C with a heated plate and heating lamps. For volume replacement and drug application the right jugular vein and for continuous blood pressure measurement, heart rate (Hewlett Packard Model 88S) and gaining blood samples the left carotid artery were canulated. The catheters were subcutaneously transferred to the occiput and further on through a small incision into a Swivel device (Instech, Plymouth, USA). This allows completely free movement with simultaneous continuously hemodynamic measurements. Afterwards median laparotomy was performed, the coecum was mobilized and grp II-V underwent standardized CLP, as had been described in detail previously by Singleton et. al [15]. In brief, 25% of the coecum was ligated and punctured twice using a sterile 18-gauge needle. Postoperatively, all rats received fluid resuscitation as follows: Animals of all groups received 0.5 ml/100 g BW/h NaCl as basal fluid therapy. Additionally, all sepsis groups received 1.0 ml/100 g BW/h NaCl (grp II), RA (grp III), Gel (grp IV) or HES (grp V) respectively (Figure 2). Analgesia was continuously provided i.v. by the following protocol: grp I: 0.25 μg/100 g BW/h fentanyl (Fagron, Germany); grp II-VI: 2.0 μg/100 g BW/h fentanyl. All animals had access to a standard diet and water ad libitum. After 24 hours rats were re-anaesthetized. A combined anaesthesia was used with A) Continuously i.v. medication: for sedation: Midazolam (Midazolam-ratiopharm®, Ratiopharm, Germany) 0.7 mg/100 g BW/h, and for analgesia: the opioid Fentanyl 7 μg/100 g BW/h and B), inhaled anaesthetics for a sufficient anaesthetic depth using isoflurane (0.6-0.8 Vol%). For a sufficient oxygenation (SaO_2_ > 93% and PaO_2_ > 60 mmHg) a tracheotomy was performed and rats were mechanically ventilated with FiO_2_ 0.28 using a Rodent Ventilator (Type: 7025, Hugo Sachs Elektronic KG, Germany). Tidal volume remained the same for all animals, if necessary breathing frequency was adjusted. For cardiac output (CO) measurements the right femoral artery was canulated and a thermocatheter (MLT1402 T-type Ultra Fast Thermocouple, ADInstruments) was introduced. CO was measured by thermodilution using 300 μl icecold NaCl and PowerLab® (ADInstruments) software. Parameters were calculated as described previously [16]. Blood gas values were measured using ABL505 blood gas analyzer (Radiometer, Copenhagen). After microcirculatory measurements or sham operation animals were sacrificed in deep anaesthesia by blood samples taken from the arterial line. Animals which died before 24 h or had MAP levels < 70 mmHG during the 24 h after CLP were excluded from the study.

### Intravital microscopy

#### Liver-microcirculation

For the realization of the intravital microscopy of the left liver lobe a transverse subcostal and median incision was performed (n = 6 for each group). The hepatic ligaments of the left liver lob were dissected and rats were placed in a 110° left position onto a modified inverted Zeiss microscope (Axiovert 200, Carl Zeiss, Göttingen, Germany). The edge of the left liver lobe was gently taken out and spread on a specific pillar. The lower surface was placed on a modified cover glass to perfuse the liver lob with 37°C warm NaCl, and heat lamps preserved body temperature. Blood perfusion within individual sinusoids was studied after contrast enhancement by fluorescein isothiocyanate-albumin (5 μg/100 g intravenously Sigma, Deisenhofen, Germany) and fluorescein sodium (1 μg/g intravenously Sigma, Deisenhofen, Germany) slow i.v. administration. The liver surface was epi-illuminated using a 100-W mercury lamp and a filter set consisting of a 450- to 490-nm excitation and a 520-nm emission bandpass filter. Than observations of the liver microcirculation were captured using different lenses (Achroplan × 10 NA0.25/×20 NA0.4/×40 NA 0.6) and digitally recorded for subsequent off-line analysis by an investigator blinded to the treatment groups. The sinusoidal diameter was assessed at sites associated with vitamin A autofluorescence linked to hepatic stellate cells as described previously [17]. At least 10 fields of view/animal were analyzed and a minimum of ten sinusoids were analysed per field of view. Details of this technique have been described elsewhere [18,19].

#### Intestinal microcirculation

In anesthetized animals, a median laparotomy was performed for the investigation of intestinal microcirculation. A part of the ileum was taken out and spread on a specific pillar without any distortion. Thereafter a incision at the anti-mesenteric side of the ileum (across from the afferent intestinal vessels) was done. One of the two halves was put on the object plate of the microscope to measure microcirculation. Suction tubes were inserted into the luminal side of the ileum to drain off the feces. Afterwards ileum tissue was kept wet and warm by 37°C NaCl-Solution and heat lamps preserved body temperature Microcirculation in at least eight villi was performed as described above. Functional Capillary Density, Velocity and Vessel-Diameter were measured after contrast enhancement by fluorescein isothiocyanate-albumin (7 μg/100 g i.v.) administration with the same lamp and filter as for the liver perfusion. For counting activated leukocytes rhodamine 6 G (1.5 mg/100 g intravenously Sigma, Deisenhofen, Germany) was administered. Then observations of the gut microcirculation were captured using different lenses [Achroplan × 20 NA 0.4 and × 40 NA 0.6 (leukocytes); 497 nm-excitation and a 524 nm-emission filter).] and digitally recorded for subsequent off-line analysis by an investigator blinded to the treatment groups.

#### Evaluation of microcirculatory flow

Velocity of erythrocytes was measured by a blinded investigator using Software MetaMorph V 6.1r4. For each point of time the velocities of 24 randomly chosen erythrocytes were measured for one image section and for each animal 3 sections were performed. For sinusoidal diameter 10 randomly chosen distances were measured through out one section. From these data volumetric flow was calculated based as described previously [20]; Volumetric blood flow (VQ) = (diameter [μm])^2^x(1/4xπ))x erythrocyte velocity [μm/ms]). Functional capillary density was measured by putting a grid across the region of interest and perfused capillaries crossing the gridlines were counted in a 30 s sequence. The value is described as capillaries per μm gridline [21].

#### Macrohaemodynamic monitoring and evaluation

Mean arterial pressure (MAP) and heart rate were continuously measured. Cardiac index (CI), stroke volume index (SVI), total peripheral resistance index (TPRI) and delivery oxygen index (DO_2_-I) were calculated as recently published using the following equations [16]: Cardiac index (CI) [ml/min/kg] = cardiac output/BW; stroke volume index (SVI) [ml/beat/kg] = CI/heart rate; Total peripheral resistance (TPRI) [mmHg/ml/min/kg] = MAP/CI; delivery of oxygen index (DO_2_-I) = (CI x (((SaO_2_ [%]/100)xHb [g/dl]x1,34) + SaO_2_[%]x0.0031)/100) while SaO_2_ = arterial oxygen saturation [%] ; Hb = haemoglobin [g/dl].

#### Markers of liver function

After vital microscopy plasma samples were drawn for determination of liver function by measuring aspartate transaminase (AST), alanine transaminase (ALT), alkaline phosphatase (ALP), total bilirubin (TBIL), gamma glutamyltranspeptidase (GGT), albumin, glucose, INR, PTT, haemoglobin (Hb) and platelet count by using routine laboratory methods.

#### Cytokines

Blood was drawn at the end of experiment, centrifuged at 3400 g for 10 min and 4°C. Afterwards samples were frozen at −80°C. For quantification a commercial available solid phase sandwich immunoassay for simultaneously quantifying multiple biomarkers with the Luminex®-method (‘Rat 10-Plex’ Invitrogen, Karlsruhe, Germany) was used according to the manufacturer’s recommendations. Analyses were done in triplicate with a Luminex®100 intrument (Luminex Corporation, Austin, Texas, USA).

#### Heamoxygenase-1 (HO-1)

Liver HO-1 was analyzed in triplicate by using standardized western blotting as previously described (HO-1 (Hsp32) assaydesigns, Lörrach, Germany) [22].

#### Statistical analysis

Values are expressed as mean ± standard error. Possible differences were assessed using SPSS 19.0 statistical software. Statistical significance was determined by analysis of variance (multivariate ANOVA followed by post-hoc Duncan test, or for nonparametric values Kurskal-Wallis test following Mann–Whitney-U Test and Bonferroni correction) or by Students t-test to determine differences in mean between the groups. Statistical significance is assumed for p < 0.05.

## Results

### Kaplan maier survival plot

**Figure 3:**
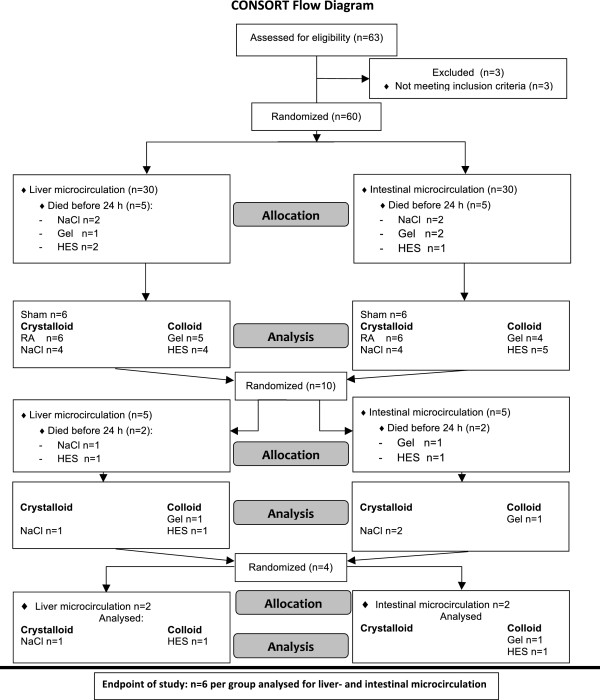
**CONSORT flow diagram.** Kaplan meier survival plot. Gel treated animals revealed highest mortality (29%) followed by HES (28%) and NaCL (28%), whereas all RA and control rats survived.

Overall sepsis mortality rate was 22%, whereas all sham operated animals survived with no signs of sepsis during the experiment. All animals infused with the crystalloid RA survive and NaCl (33%), HES (33%) and Gel (29%) exhibited reduced survival rates (Figure 3). Septic animals displayed, with no differences between the groups, clinical features of illness from about 1–2 h after CLP procedure, including a decrease in activity, reduced alertness, ruffled fur, and hunched posture. This clinical status deteriorated continuously over time. Control animals appeared outwardly normal.

### Microcirculation

#### Liver

**Figure 4:**
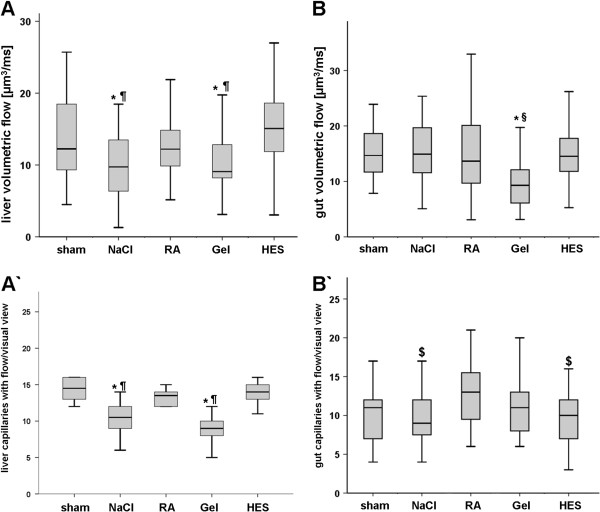
**Microcirculation.** Liver microcirculation 24 h after CLP. NaCl and Gel revealed significantly decreased volumetric flow (**A**) and a reduced amount of liver capillaries with flow (**A’**) compared to HES. A)Intesinal microcirculation 24 h after CLP. Gel treated animals showed significant impairment of volumetric flow compared with all other groups (**B**). The number of perfused capillaries was significantly increased in RA treated animals when compared with NaCl and HES (**B’**). n = 6 animal/ group; p < 0.05, mean ± SD; * vs. HES, § vs NaCl and RA, $ vs. RA.

Despite of the increased mortality in the colloid groups, volumetric flow [μm^3^/ms] of 6% HES 130/0.4 (15.3 ± 5.6) was significantly improved compared to Gel (10.4 ± 3.9) and NaCL (10.2 ± 6) (Figure 4A). However no significant difference between RA (12.5 ± 3.6) and HES was detectable. Sinusoidal diameters were significantly reduced as a sign of inflammation in all septic animals (NaCl 7.5 ± 1.7; RA 7.7 ± 2; Gel 7.6 ± 2.3; HES 7.5 ± 2.4 [μm]) compared to sham (8.3 ± 1.5 [μm]). Capillary density was significantly reduced in NaCl and Gel treated animals, whereas HES and RA showed no differences compared to sham (Figure 4A’). But all sepsis groups revealed more vessels with slow velocity as a marker of impaired microcirculation (data not shown).

#### Intestine

Volumetric blood flow [μm^3^/ms] was significantly lower in Gel (11.1 ± 6.1) than in all other groups (Sham (15.9 ± 7.2), NaCl (16.9 ± 9.3), RA (14.9 ± 8.0), HES(15.2 ± 5.0); Figure 4B). The number [n] of capillaries with flow were significantly increased in the RA group (12.6 ± 3.7) compared to NaCl (9.9 ± 3.5) and HES (9.6 ± 3.5); Figure 4B’). Intestinal vessel diameters were only significantly increased in HES animals (6.6 ± 2.3 [μm]) when compared to all other groups NaCl (6.0 ± 1.7), RA (6.1 ± 2.1) and Gel (6.2 ± 2.1). The number of moving leucocytes was significantly higher in the Gel group than in HES and RA. The total number [n] of activated leucocytes (sticking and rolling) was significantly higher in Gel (4.4 ± 3.2) compared to RA (2.5 ± 2.0) and HES (2.4 ± 1.9) (data not shown).

#### Macrohemodynamics and blood gas analyses

MAP, CVP, HR, pH and p_a_CO_2_, showed no significant differences between the groups over time. CI [ml/min/kg] was significantly increased in the colloid groups (HES 541 ± 60; Gel 481 ± 113) when compared with CL treated animals. But SVI [ml/beat/min] did not significantly differ between the CL and COL treatment groups. P_a_O_2_ showed no differences between the groups, where as DO_2_-I improved significantly in HES infused animals compared to all other septic groups (Tables 1 &2). TPRI [mmHg/ml/min/kg] was reduced only in HES (0.17 ± 0.1) infused animals. Hb [g/dl] was significantly reduced in Gel (8.7 ± 1.3) and HES group (11.4 ± 1.4) when compared to sham (13.9 ± 0.8; Table 1). NaCl and Gel group revealed metabolic acidosis represented as significantly reduced pH, HCO_3_^-^, SBE (Table 2). All other septic animals demonstrated also derangements in metabolic state but reached no significance due to elevated standard deviation.

**Table 1:** Evaluation of macrohemodynamic parameters after mechanical ventilation and 24 h CLP or sham

**Groups**	**paO**_**2**_	**SaO**_**2**_	**Hb**	**CI**	**HR**	**SVI**	**MAP**	**DO**_**2**_**-I**	**TPRI**
	**[mmHG]**	**[%]**	**[g/dl]**	**[ml/min/kg]**	**[/min]**	**[ml/beat/min]**	**[mmHG]**	**[ml/min/kg]**	**[mmHg/ml/min/kg]**
Sham	124 ± 28	96 ± 2	13.9 ± 0.8	355 ± 60	411 ± 48	0.87 ± 0.13	101 ± 22	64,6 ± 11	0.29 ± 0.06
NaCL	119 ± 44	95 ± 2	12.6 ± 1.7	332 ± 162	405 ± 40	1,04 ± 0.39	91 ± 23	69,7 ± 38	0.23 ± 0.10
RA	94 ± 42	90 ± 9	12.4 ± 1.9	374 ± 150	439 ± 40	0.85 ± 0.33	98 ± 18	54,4 ± 18.8	0.29 ± 0.12
Gel	115 ± 42	92 ± 9	8.7 ± 1.3*	481 ± 113*	437 ± 55	1.1 ± 0.24	91 ± 17	52,6 ± 13.9	0.20 ± 0.07
HES	119 ± 43	96 ± 2	11.4 ± 1.4*	582 ± 85^#^*	429 ± 37	1.36 ± 0.21*	96 ± 20	88,6 ± 21*	0.17 ± 0.10*

(cardiac index (CI), stroke volume index (SVI), delivery oxygen index (DO_2_-I), total peripheral resistance index (TPRI); p < 0.05, *vs sham,# vs RA and NaCl.

**Table 2:** Blood gas analyses measured 30 min after surgical procedures (baseline) and after intravital microscopy

**Baseline**	**pH**	**paCO**_**2**_**[mmHG]**	**HCO**_**3−**_**[mmol/l]**	**SBE**_**−**_**[mmol/l]**	**24 h CLP**	**pH**	**paCO_2_ [mmHG]**	**HCO_3−_ [mmol/l]**	**SBE_−_[mmol/l]**
Sham	7,47 ± 0,05	38 ± 6	27,1 ± 1,5	3,5 ± 1,6	Sham	7,40 ± 0,03	39 ± 7	23,7 ± 3,8	−0,2 ± 3,4
NaCl	7,4 ± 0,02	44 ± 4	26,7 ± 2,0	2,3 ± 2,0	NaCl	7,29 ± 0,15	37 ± 10	20,2 ± 4,2*	−6,5 ± 4,8
RA	7,44 ± 0,02	41 ± 3	28,7 ± 4,6	3,4 ± 1,0	SteroIso	7,37 ± 0,05	41 ± 6	24,7 ± 3,8	−1,4 ± 3,5
Gel	7,42 ± 0,04	39 ± 6	25,6 ± 2,5	1,1 ± 2,7	Gel	7,26 ± 0,11*	33 ± 12	15,1 ± 5,9*	−10,7 ± 6,7*^$^
HES	7,41 ± 0,04	45 ± 6	27,9 ± 4,1	2,8 ± 1,7	HES	7,37 ± 0,03	37 ± 3	21,3 ± 2,2	−4,6 ± 5,7

p < 0.015-0.05, * vs. sham;$ vs. RA.

#### Cytokines

**Figure 5:**
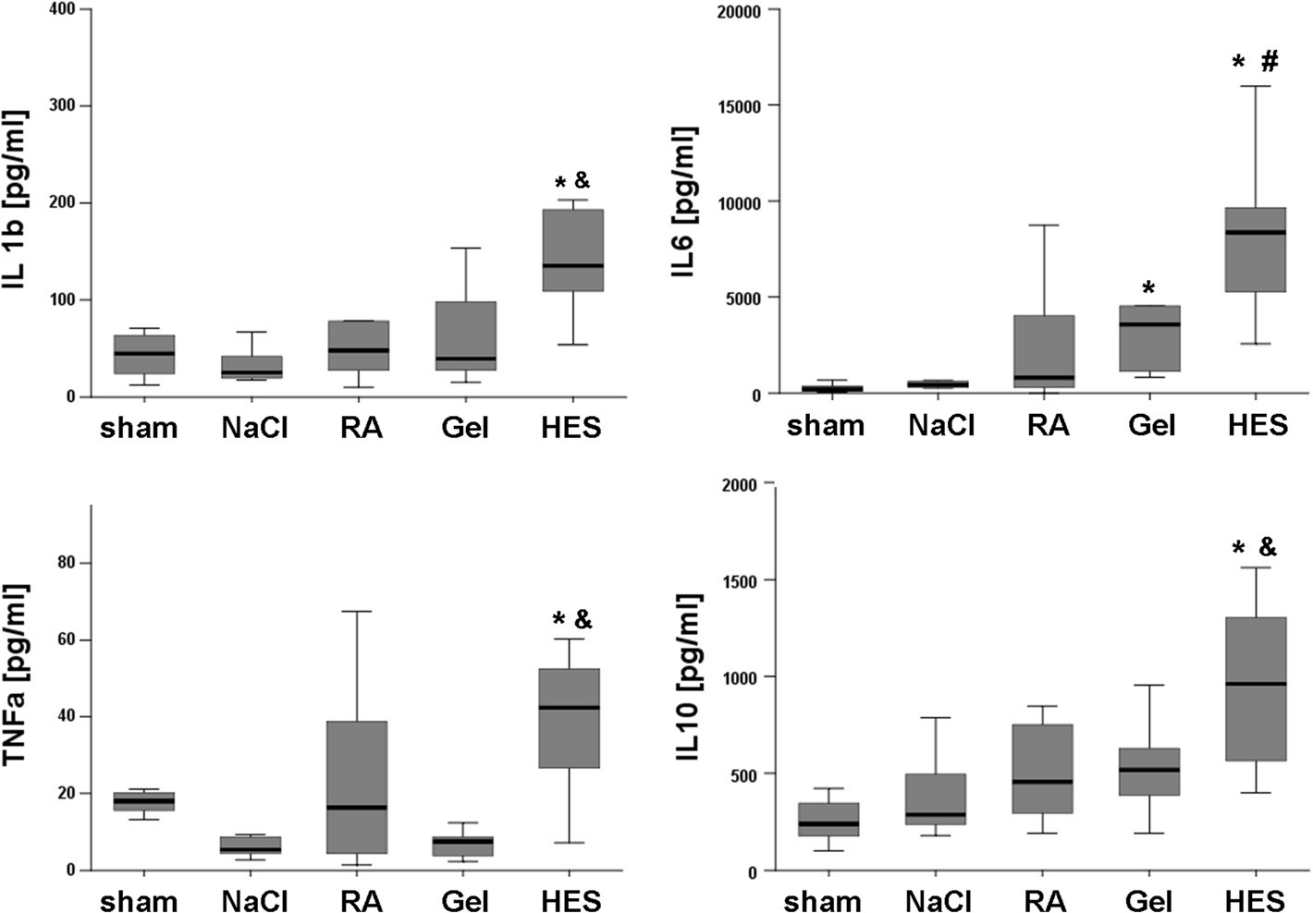
**Cytokines.** Box-and-whisker plots showing cytokine levels of the serum 24 h after CLP. HES animals showed increased levels of cytokines when compared with other septic animals. The boxes represent the 25^th^ to 75^th^ percentiles, and horizontal lines within the box represent median values. The whiskers represent the lowest and highest value in the 25^th^ percentile minus 1.5IQR and 75^th^ percentile plus 1.5IQR regions; n = 10-12 per group; p < 0.05; * vs. sham, & vs. Gel, RA and NaCl, # vs. RA and NaCl.

IL-1b, IL 10 and TNFα showed significantly elevated levels in the HES group when compared with crystalloids. IL-6 levels were significantly higher in the Gel- and HES-group (Figure 5).

#### Liver injury and function

##### Injury markers

The biomarkers for liver injury AST, AP as well as HO-1 did not show any difference between all groups, but AST tended to be increased in all septic animals and Gel showed the highest level. ALT [U/l] was significantly increased in the Gel (341 ± 423) and NaCl (168 ± 117) group compared to sham (55 ± 12, Table 3).

**Table 3:** Liver parameters measured at the end of experiment

**Group**	**AP**	**AST**	**ALT**	**GGT**
	**(U/l)**	**(U/l)**	**(U/l)**	**(U/l)**
Sham	128 ± 43	162 ± 64	55 ± 12	1,9 ± 2,1
NaCl	117 ± 41	258 ± 207	168 ± 117*	3,5 ± 2,5
SteroIso	151 ± 58	249 ± 81	105 ± 41	5,8 ± 3,5
Gel	147 ± 51	427 ± 361	341 ± 423*	5,1 ± 3,4
HES	163 ± 72	297 ± 246	142 ± 48	5,8 ± 5,1

p < 0.05, mean ± SD, *vs sham.

#### Liver function

**Figure 6:**
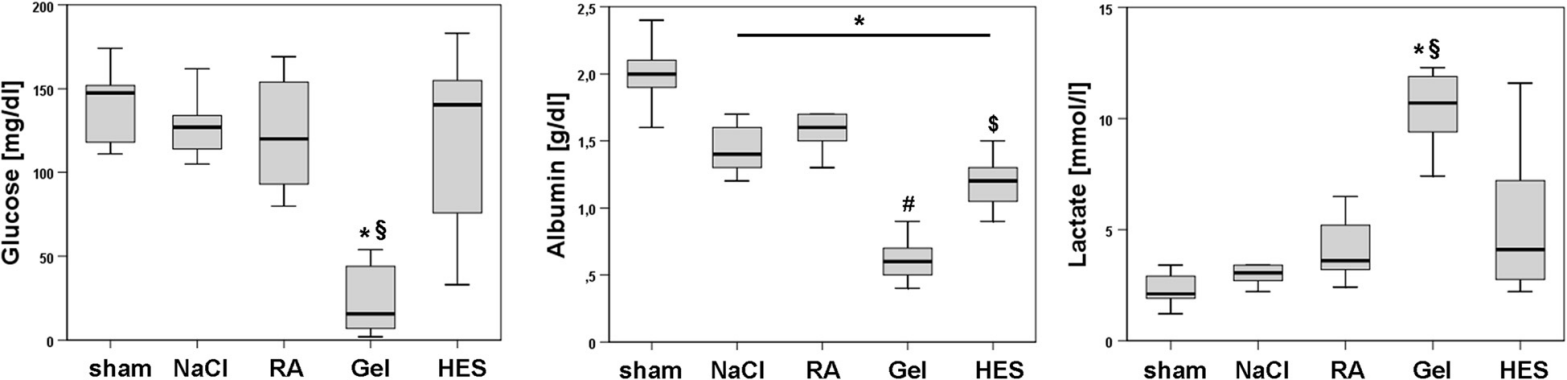
**Liver function.** Box-and-whisker plots showing functional liver parameters revealed severe side effects of gelatine infusion. Glucose level was significantly reduced compared to all other groups. Albumin was decreased in all septic animals, but Gel showed the lowest level compared to all other groups. Kolloids showed significant increased lactate levels and Gel revealed the highest level. Parameters were determined at the end of the experiment. The boxes represent the 25^th^ to 75^th^ percentiles, and horizontal lines within the box represent median values. The whiskers represent the lowest and highest value in the 25^th^ percentile minus 1.5IQR and 75^th^ percentile plus 1.5IQR regions; * vs. sham; § vs. NaCl, RA and HES; p < 0.05. n = 10-12 per group.

The investigation of liver function revealed a significant reduction of albumin in all septic rats and gelatine treated animals showed the lowest albumin levels compared with all other investigated groups. Colloids showed increased lactate levels whereas Gel treated animals revealed the highest levels. Gel [mg/dl] infused animals had significantly decreased glucose level (33 ± 44) compared with NaCl (121 ± 33), RA (124 ± 33) and HES (126 ± 50), (Figure 6).

#### Coagulation disorders

**Figure 7:**
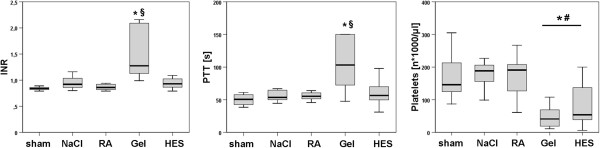
**Coagulation parameters.** Box-and-whisker plots showing coagulation analyses 24 h after CLP. Gelatine infused rats showed coagulation derangements. (The boxes represent the 25^th^ to 75^th^ percentiles, and horizontal lines within the box represent median values. The whiskers represent the lowest and highest value in the 25^th^ percentile minus 1.5IQR and 75^th^ percentile plus 1.5IQR regions; p < 0.05; * vs. sham; § vs. NaCl, RA and HES; n = 10-12.

Gelatine infusion led to significant coagulation impairments. INR (1.51 ± 0.5) and PTT (108 ± 40) were significantly increased compared to all other groups. Platelet counts [*1000/μl] were significantly decreased in the colloid groups Gel (56 ± 50) and HES (83 ± 71) when compared with crystalloids (Figure 7).

## Discussion

Volume resuscitation is a pivotal therapy in sepsis and septic shock, but the level of evidence-based recommendation of the type of intravenous fluid is still low. Furthermore the required quantity of volume to stabilize haemodynamics remains a challenge by the complex interaction of endothelial-, epithelial barrier disruption, cytokine storm, septic cardiomyopathy, hypo- or hyperthermia, cavity edema, coagulation disorders with consecutive bleeding and co-morbidity [23].

In patients suffering from endothelial barrier disruption like in sepsis, the estimated volume effect of colloids is less than expected. For example, in the Scandinavian Starch for Severe Sepsis/Septic Shock (6S) trial, patients in the HES group received almost the same amount of volume when compared with patients in the RA group to achieve hemodynamic stabilisation [13]. Silva et al. have shown recently in rats with CLP-induced sepsis that an infusion of 6 ml/100gBW/h with gelatine resulted in hypervolemia [21]. The right colloid to crystalloid ratio is still an open question, and we therefore decided to use a 1:1 ratio to investigate the same amount of different infusion solutions. Furthermore our fluid regime comprises two sections: 1) 0.5 ml/100gBW/h NaCl: to cover the normal fluid intake of rats and 2) additional we administered 1.0 ml/100gBW/h of the different solutions to compensate fluid loss of surgical procedures and sepsis (Figure 2). Singelton et al. showed a 40% mortality rate in the first 24 h and a significant elevated IL-6 level, when 25% of the coecum was ligated and punctured twice [15]. We established this model in our lab to achieve severe sepsis and a reduction of lethality by sufficient analgesia and volume resuscitation. The aim of the protocol was to get a fast (within 24 h) onset of beneficial and/or adverse side effects of fluid solutions in septic rats, and not a “patient” adapted goal directed fluid resuscitation trial. The degradation of starch molecules or the metabolic state of rats are two of numerous differences between rodents and humans and conclusions from animal models to clinical practice should be done with caution. Most notably, our rodent model, investigating 24 hours after the CLP procedure, reflects the early stage of severe sepsis. Patients in the clinical setting are often treated in the later phases of severe sepsis.

However, the pathomechanism of septic liver failure is poorly understood and it is meanwhile known, that macrohemodynamic parameters do not reflect microcirculation and microcirculation by itself differs extensively between different organs, even when systemic macrohemodynamic steady state values were achieved. Little is known about the impact of different fluid solutions on different intestinal organs, and there is still an ongoing discussion about the ideal fluid solution in sepsis. The scepticism about the benefit of synthetic colloids in volume therapy is increasing [10,12,24]. Colloid solutions should have a greater volume effect than crystalloids. Indeed, the colloid group had a significantly increased cardiac index, but only the HES solution resulted in an increased stroke volume index (Table 1). Despite of significantly reduced Hb levels, HES showed improved oxygen delivery, but other macrohemodynamic parameters, such as HR, MAP and CVP, did not show any differences between the septic groups (Table 1). Furthermore HES infused rats had a decreased peripheral resistance, and in line only HES showed dilatation of the intestine vessels. Additional HES improved volumetric flow of the liver sinusoids compared to Gel, NaCL and HES had no decreased microcirculation and number of capillaries under flow (CUF) in the liver compared to sham (Figure 4). Interestingly only gelatine revealed impaired gut microcirculation, whereas HES showed no such effect. RA revealed increased number of CUF and did not impair volumetric flow of the gut. The different patterns of liver and intestine microcirculation in the septic groups underline, that different organ react differently to sepsis and volume resuscitation. Taken together, 6% HES 130/0.4 revealed a higher volume effect, increased DO_2_-I and showed improvement of liver microcirculation and RA might be benefical for the gut and liver microcirculation. But the improvements in the starch group did not correlate with survival. All septic animals showed the clinical signs of severe sepsis and revealed an overall mortality rate of 22%. HES, Gel and NaCL infused animals revealed mortality around 30%, whereas the RA group survived entirely. We have shown previously, that HES and Gel impair kidney function in rodent sepsis [12]. COL solutions resulted in functional and histological impairments when compared with CL solutions. However, the effects of the different solutions on the kidney may be causative for the mortality differences between the groups.

RA was the only balanced solution in this trial, but clinical data about the influence of balanced solutions on mortality of patients remains heterogeneous. It has been stated recently by Guidet et al., that balanced crystalloid solutions do not show any effect on mortality of patients when compared with isotonic saline [25]. In our study, animals treated with NaCl and Gel showed severe acidosis. HES treated animals, with NaCl as a part of the HES solution and saline as baseline infusion showed no deranged acid base status. However the acid–base equilibrium or the presence of a dilutional-hyperchloraemic acidosis was not the focus of this survey.

Gelatine, which is often described as safe and harmless, developed acidosis, which may be due to reduced Hb following anaerobic metabolism, and additionally Gel group showed severe hypoglycaemia which may increase lactate and aggravated metabolic acidosis. The acidosis may be the reason for the coagulopathy, but NaCl treated animal revealed also reduced pH-level and had no derangements in the coagulation parameters. However, Gelatine significantly impaired coagulation, represented by elevated PTT, INR, decreased platelet count, as well as clinical signs of coagulopathy (hematothorax, intraabdominal bleeding). This may be caused by a direct effect of gelatine on the coagulation system and on the liver coagulation factor synthesis. It is well known that artificial plasma expanders may influence the coagulation system more than crystalloids in-vitro and in-vivo beyond the effect of dilution [26]. In addition, colloids could be stored by the reticulo endothelial system (RES) and may lead to impaired phagocytic activity particularly in septic shock [27]. Reduced phagocytic properties of the RES lead to decreased elimination of activated fibronectin, which can be stimulated by collagenous structures such as gelatine. Thus the reduction of elimination might be another explanation for the activated coagulation system by gelatine in septic rats, which may lead to dissiminated intravascular coagulopathy.

Vlahos et al. demonstrated that CLP impaired hepatocytes exocrine synthesis, and incubation with colloids (albumin or HES) had a direct and dose depended impact on albumin and urea production [28]. Furthermore CLP pre-treated hepatocytes developed severe morphological cell injury due to colloids despite metabolic activity in vitro. Thus significantly increased level of ALT and elevated AST in Gel group may be explained by acute liver damage. However, despite of decreased microcirculation, decreased sinusoidal diameter and impaired liver function histopathology investigation did not show any differences between the groups. This may due to the short investigation period of 24 h. In opposite to synthetical colloids RA showed no coagulopathy or liver derangements.

It has been shown in the literature, that HES exhibits an anti-inflammatory effect in different organs and species. The main differences with our experiment are the amount and incubation time of HES. Lv et al.showed an anti-inflammatory response of HES compared to CL, but they compared 6.4 ml/100gBW crystalloids up to 1,6 ml/100gBW HES within one hour infusion regime in rats [29]. Thus the CL group might be hyperinfused and the consecutive hypervolemia increased cytokine levels itself. However, this infusion strategy (COL to CL ratio of 1:4) was the golden standard. We could show, that 24 h high colloid exposition lead to a significant increase of pro-inflammatory cytokines such as TNF-α, IL-1β and IL-6 in HES group.

## Conclusion

The balanced crystalloid infusion RA could stabilize micro- and macrohemodynamic, had least side effects and all animals survived when used in septic rats. 6% HES 130/0.4 improved cardiac output, DO_2_-I and microcirculation of the liver, but showed elevated IL-1β, IL-6 and TNF-α levels and a mortality rate of 33%. Gelatine 4% revealed severe coagulopathy, hypoglycaemia, elevated liver enzymes, impaired microcirculation and a 29% lethality. Taken together, in our rodent model of CLP-induced severe sepsis, crystalloid infusion revealed best results in mortality and microcirculation when compared with colloid infusion.

## Competing interests

The authors declare that they have no competing interests.

## Authors’ contributions

MAS contributed to animal preparation, performance of experimental work, analysis of mechanical and histological data, statistical analysis, and writing of the manuscript. JTI contributed to animal preparation, performance of experimental work, preliminary data analysis, and drafting of the manuscript. TS contributed to performance of experimental work, analysis of mechanical and histological data and statistical analysis. JB contributed to analysis of data and drafting of the manuscript. JS contributed to preliminary data analysis, and drafting of the manuscript. NS contributed to performance of experimental work and analysis of data. NR contributed to experimental design and writing of the manuscript. OE contributed to the experimental design. CW contributed to experimental design, supervision of experimental work, statistical analysis, writing of the manuscript, and supervision and overview of entire project. All Authors read and approved the final manuscript.

## Pre-publication history

The pre-publication history for this paper can be accessed here:

http://www.biomedcentral.com/1471-230X/12/179/prepub
